# The Association Between Family Environment and Adolescent Alcohol Drinking Behavior: A Cross-Sectional Study of Six Chinese Cities

**DOI:** 10.3389/fnut.2022.903216

**Published:** 2022-06-14

**Authors:** Ruixin Chi, Shijun Lu, Na Zhang, Man Zhang, Kaiwei Guo, Songming Du, Jing Guo, Xiaoqi Hu, Guansheng Ma

**Affiliations:** ^1^Department of Nutrition and Food Hygiene, School of Public Health, Peking University, Beijing, China; ^2^Laboratory of Toxicological Research and Risk Assessment for Food Safety, Peking University, Beijing, China; ^3^Institute of Food and Nutrition Development, Ministry of Agriculture and Rural Affairs, Beijing, China; ^4^Chinese Nutrition Society, Beijing, China; ^5^Epidemiology Directorate, Department of Health WA, Perth, WA, Australia; ^6^National Institute for Nutrition and Health, Chinese Center for Disease Control and Prevention, Beijing, China

**Keywords:** family environment, alcohol drinking (MeSH), family, adolescent, underage drinking

## Abstract

**Objective:**

Adolescents' alcohol consumption has lifetime adverse physical and mental health effects. Family environment factors have a significant influence in shaping adolescents' beliefs and habits. We conducted the multicenter cross-sectional study aiming to investigate the association between family environment factors and adolescent drinking behavior in China.

**Methods:**

The study investigated 27,762 middle school students from Beijing, Shanghai, Guangzhou, Jinan, Chengdu, and Harbin. A logistic regression model was used to explore the association between family environmental factors and adolescent drinking behavior. Participants were asked to self-report previous experiences of drinking and getting drunk to access their drinking status. Factors of family environment related to alcohol consumption included: parents' educational level, family economic status, family composition, the number of times parents drank alcohol in the past 30 days, and parents' attitudes toward their drinking behavior. The logistic regression model was used to adjust the demographic confounders, including gender, age, city, location, and smoking status, and to explore the association between family environmental factors and adolescent alcohol drinking behaviors.

**Results:**

Compared with students whose parents prohibited drinking, students who were approved drinking were more likely to drink in this year (*OR* = *16.544, 95%CI:15.265–17.929, P* < *0.001*; Full adjustment: *OR* = *13.111, 95% CI: 12.031–14.288, P* < *0.001*), drink in this month (*OR* = *7.791, 95% CI: 7.077–8.565, P* < *0.001*; Full adjustment: *OR* = *6.010, 95% CI: 5.439–6.641, P* < *0.001*). In addition, Low family economic status, not living with the mother, parents' ambivalent attitudes toward their children's drinking and parental drinking were risk factors for drinking among middle school students.

**Conclusion:**

The family environment, especially parents' attitudes, is associated with students' drinking and drunken behavior. Mobilizing the power of parents may play a positive role in the effective prevention and control of adolescent drinking.

## Introduction

Alcohol is harmful to people's health. Worldwide, alcohol consumption is responsible for nearly three million deaths in a year. Alcohol is a significant risk factor for premature death and disability in people aged 15–49. Meanwhile, alcohol has toxic effects on the central nervous system, digestive system, and cardiovascular system, and acts as an immunosuppressant to increasethe risk of infectious diseases (1).

For adolescents, alcohol use could lead to long-term health consequences. Adolescents are at an important stage of growth and development; and this stage has an important impact on the development of the brain's structure and function. Alcohol intake can cause changes in the brain, causing impaired neurodevelopment, affecting cognitive and behavioral functions, and resulting in the decline of academic performance and frequent injury events (2). Adolescent alcohol consumption impairs physical and mental health during adolescence and throughout lifetime (3, 4). A global study showed that alcohol (7% of Disability-adjusted life years) was one of the significant risk factors affecting people's (age from 10 to 24) health (5). This findings suggest public health strategies should focus on children and adolescents and prioritize adolescent limiting alcohol consumption (6).

Alcohol is prevalent among teenagers all over the world, including Chinese teenagers. WHO(World Health Organization) reported that 43.0% of people over 15 years old worldwide are drinking, and 12.5% have been drinking before (1). According to a cross-sectional survey conducted in southern China, the current alcohol consumption rate among 9–21 years old is 7.3% (7). A 2005 survey of youth risk behaviors in 18 provinces and cities in China showed that about 51.1% of young people in grades 7 to 12 had used alcohol before (8). A study in the United States showed that 78.2% of teenagers drink alcohol in late adolescence. And the rate of drinking is increasing year by year (9). It is of great importance to pay attention to adolescents' drinking behavior, to understand the factors affecting adolescent drinking, and to control them for improving the health of the adolescent (6).

It has been proved that the food environment can affect adolescents' eating behaviors, such as fruit and vegetable intake, calorie intake, fat intake, and water drinking behavior (10–14). Some studies have also attempted to explore the influence of family environment on adolescent drinking behavior (15, 16). Family composition (17, 18), family economic status (19), parents' educational level (20), drinking behavior (21–24), and parents' attitude (19) toward their children were all mentioned (25). However, the results were inconsistent across different studies. Moreover, variables related to the family environment are not sufficient in studies.

Current research evidence is insufficient to show the relationship between family environment and adolescent drinking behavior in China. This study attempted to investigate the association between family environment and adolescent drinking behavior in China.

## Methods and Materials

### Participants

The study was performed in middle school students in China. We carried out a cross-sectional survey in Beijing, Shanghai, and Guangzhou in 2013 and completed the survey in Jinan, Chengdu, and Harbin with the same process in 2014. This study used a multi-stage stratified cluster sampling method to select 4–6 urban districts in each city based on administrative division, geographical location, and class size. Middle and high schools (including ordinary high schools and vocational schools) were selected from the selected urban districts. There were three grades in both middle and high schools in China. If it were impossible to investigate the third-grade class due to the college entrance examination or internship, we would select one more second-grade class from the school for investigation. Participants completed a voluntary and anonymous self-management questionnaire during class. Subjects were informed of the purpose of the study and will have the right to refuse to participate. Students who complete the questionnaire will be rewarded with gifts.

### Ethics Statement

The Ethics Committee of the National Institute for Nutrition and Food Safety, Chinese Center for Disease Control and Prevention, approved the study. The ethical approval number is 2013–19. All investigations were conducted with the informed consent of all participating students, guardians, and school units.

### Demographic Characteristics

Demographic characteristics were measured with a questionnaire. Participants reported their gender, age, cities, and location. The general information questionnaire was used to collect basic demographic information, including gender, age, city, location, and whether the study subjects smoked. Locations were divided into urban and suburban areas.

### Assessment of Alcohol Drinking Behaviors

According to the Youth Risk Behavior Survey (YRBS) questionnaire and Global School-based Student Health Survey (GSHS) of WHO, the questionnaire of Drinking Behavior of Middle School Students used in this survey was determined through the discussion of experts of the project team. The subjects were asked to fill in the questionnaire according to their actual situation. A detailed analysis of drinking behavior will be carried out separately. In this study, only the following indicators were included: “whether you ever consumed alcohol,” “whether you consumed alcohol within a year,” “whether you consumed alcohol within a month,” and “whether you were ever drunk.” The outcome variables were all binary variables, divided into “yes and no. The questionaires used in this survey was determined through the discussion of experts of the project team. The subjects were asked to fill in the questionnaire according to their actual situation. A detailed analysis of drinking behavior will be carried out separately. In this study, only the following indicators were included: “have you ever consumed alcohol,” “have you consumed alcohol within a year,” “have you consumed alcohol within a month,” and “have you ever being drunk.” The outcome variables were all binary variables, divided into “yes” and “no.”

### Family Environment Related to Drinking

In this study, family environment variables associated with alcohol consumption included: family economic status, father's education level, mother's education level, and cohabitants. In addition, the times of parents' drinking behaviors and attitudes toward student alcohol consumption were also taken into consideration. We used the family affluence scale (FAS) to measure family economic status described in our previous study (26). Cohabitants fall into four categories: living with both parents, living only with their father, living only with their mother, and living with others. Parents' education level was divided into three categories, high school and lower, college, and graduate school. The number of times parents drank alcohol was defined as never, 1–4 times a month, or more than 4 times a month. The scale measuring parental attitudes favorable toward drinking includes three options, including “forbid” (0), “agree” (1) and “not sure” (2).

### Statistics

We used SAS 9.4 and SPSS 25 for data cleaning and analysis. For questions with logical errors, according to the cleaning principle of CDC in the YRBSS survey, only the questions with logical errors were set as missing, but the whole record of the investigator was not deleted, and the result analysis of other questions was not affected. Logistic regression analysis was used to analyze the relationship between family environment and adolescent drinking behavior. We set drinking behavior as a dependent variable. We included variables related to family environment, including family economic status, father's education level, mother's education level, cohabitant, parents' alcohol behaviors, and attitudes. Confounders were determined by literature review and expert discussion, including demographic characteristics and smoking status (27). In Model 2, we adjusted common counfounders of alcohol drinking, including age, gender and smoking. The study included data from 6 different cities. The drinking habits themselves from these regions can be profoundly different, so we also adjusted city and location in this model. The adjusted model was named Model 2. The difference was statistically significant at *P* < 0.05.

### Quality Control

Before the survey, all investigators were provided with a standard training manual and a workshop to explain the survey. The investigators were made up of medical workers with experience in epidemiological investigations, educators, and staff from local Disease Control and Prevention centers. The questionnaire results were filled in by students using an answer sheet that could be scanned by an automatic laser card reader. After scanning, two people check the data and finally save the data in excel form.

## Results

### Demographic Information

The total number of participants was 27,762, including 13,417 males (48.3%) and 14,345 females (51.7%). The age distribution of subjects ranged from 12 to 20 years [*M (IQR)* = 15.00 (3)]. The subjects from Beijing, Shanghai, Guangzhou, Jinan, and Chengdu were 3,782, 3,142, 5,241, 4,889, 5,363, and 5,345. Accounting for 13.6%, 11.3%, 18.9%, 17.6%, 19.3% and 19.3%, respectively. 14,375 (51.8%) of the subjects came from urban areas and 13,387 (48.2%) from suburban areas. The general characters of this study participants are shown in Table 1.

**Table 1:** The demographic information of participants.

		***N***	**%**
**Gender**			
	Male	13417	48.3
	Female	14345	51.7
**Age**			
	12~14	10234	36.9
	15~17	14516	52.3
	18~20	3012	10.8
**City**			
	Beijing	3782	13.6
	Shanghai	3142	11.3
	Guangzhou	5241	18.9
	Jinan	4889	17.6
	Chengdu	5363	19.3
	Harbin	5345	19.3
**Location**			
	Center	14375	51.8
	Suburb	13387	48.2
**Total**		27762	100.0

### Alcohol Drinking Behavior

The results show that 15,723 students (56.6%) had drunk alcohol before; 10,925 students had drunk alcohol in the past year, accounting for 44.8%; 6107 students had drunk alcohol in the past month, accounting for 23.3%. Four thousand nine hundered fivety students had been drunk, accounting for 19.3%. Among the respondents, 61.4% of male and 52.2% of female ever drank alcohol. Over the past year, 51.8% of men and 38.1% of women ever drank alcohol. 29.0% of men and 18.1% of women ever drank alcohol over the past month. And 23.6% of men and 15.3% of women ever been drunk. There were statistically significant differences in alcohol-related behaviors among subjects of different genders (*P* < *0.05*). Among the subjects aged 12 to 14, 15 to 17, and 18 to 20, the drinking rates were 32.8%, 62.9%, and 73.2%, respectively. The proportion of those who had drunk in the past year was 30.6%, 51.5%, and 61.6%, respectively. The proportion of people used alcohol in past month was 16.0%, 26.8%, and 31.8%. The rates of previous drunkenness were 9.6%, 23.3%, and 33.7%, respectively. There were statistically significant differences in alcohol-related behaviors among subjects of different ages (*P* < *0.05*). In Beijing, Chengdu, Guangzhou, Harbin, Jinan, and Shanghai, those who drank alcohol were 50.8%, 64.2%, 66.6%, 49.6%, 52.9%, and 51.9%, respectively. The proportion of people who had drunk alcohol in the past year was 37.3%, 50.2%, 54.1%, 39.8%, 41.8%, and 42.2%, respectively. The proportion of people who had drunk alcohol in the past month was 24.8%, 23.9%, 26.5%, 19.7%, 23.4%, and 21.2% and the proportion of people who had been drunk was 19.1%, 23.4%, 21.1%, 18.8%, 16.2%, and 15.1%, respectively. There was a significant difference in the rate of alcohol drinking-related behaviors among the subjects in different cities (*P* < *0.05*). In urban and suburban areas, the proportion of people who had drunk alcohol ever was 57.7% and 55.4%, the proportion of people who had drunk alcohol in the past year was 46.0% and 43.5%, the proportion of people who had drunk alcohol in the past month was 24.7% and 21.8%, and the proportion of people who had drunk alcohol in the past month was 18.4% and 20.2%, respectively. There were statistically significant differences in alcohol-related behaviors between urban and suburban subjects (*P* < *0.05*). Participates' alcohol drinking behaviors are shown in Table 2.

**Table 2:** Participates' alcohol drinking behaviors.

		**Ever consumed** **alcohol**	**Consumed alcohol** **within a year**	**Consumed alcohol** **within a month**	**Ever drunk**
**Gender**					
	Male	61.4%	51.8%	29.0%	23.6%
	Female	52.2%	38.1%	18.1%	15.3%
**Age**					
	12~14	32.8%	30.6%	16.0%	9.6%
	15~17	62.9%	51.5%	26.8%	23.2%
	18~20	73.2%	61.6%	31.8%	33.7%
**City**					
	Beijing	50.8%	37.3%	24.8%	19.1%
	Shanghai	64.2%	50.2%	23.9%	23.4%
	Guangzhou	66.6%	54.1%	26.5%	21.1%
	Jinan	49.6%	39.8%	19.7%	18.8%
	Chengdu	52.9%	41.8%	23.4%	16.2%
	Harbin	51.9%	42.2%	21.2%	15.1%
**Location**					
	Center	57.7%	46.0%	24.7%	18.4%
	Suburb	55.4%	43.5%	21.8%	20.2%
**Total**		56.6%	44.8%	23.3%	19.3%

### Family Environment

Table 3 shows the family environment related to drinking among the subjects. Most of the subjects' parents had a secondary school education or below. Most of the subjects lived with their parents. 77.1% of the study subjects reported that their fathers did not drink alcohol, and 78.9% reported that their mothers did not drink alcohol. Parents who forbid, support, and unsure about their children's drinking accounted for 39.9%, 47.3%, and 12.7%, respectively.

**Table 3:** Family environment about alcohol drinking.

		***N***	**%**
**Father's education level**	
	Secondary and lower	19621	77.1
	University degree	4969	19.5
	Graduate degree	845	3.3
**Mather's education level**	
	Secondary and lower	20028	78.9
	University degree	4729	18.6
	Graduate degree	620	2.4
**Cohabitant**			
	Parents	20110	72.5
	Father	2554	9.2
	Mother	942	3.4
	Other	4113	14.8
**Father's drinking frequency**
	0 times	7261	77.1
	1~4 times/month	9605	19.5
	> 4 times/month	7206	3.3
**Mother's drinking frequency**
	0	18853	78.9
	1~4 times/month	6085	18.6
	> 4 times/month	788	2.4
**Parental attitudes**	
**favorable toward drinking**	
	Forbid	10829	39.9
	Agree	12835	47.3
	Not sure	3445	12.7
**FAS**			
	Low	7107	26.3
	Middle	13646	50.5
	High	6285	23.2
**Total**		22762	100.0

### The Association Between Family Environment and Adolescent Drinking Behavior

The results demonstrate that family environment was closely related to adolescent drinking behavior. The association between family environment and adolescent drinking behavior is shown in Table 4. Subjects whose parents supported their drinking were more likely to drink alcohol (ever consumed alcohol (*OR, odds ratio*= *15.069, 95% CI, confidence interval:13.998–16.222, P* < *0.001*; Full adjustment: *OR* = *12.741, 95% CI: 11.772–13.789, P* < *0.001*), consumed alcohol in this year (*OR* = *16.544, 95% CI:15.265–17.929, P* < *0.001*; Full adjustment: *OR* = *13.111, 95% CI: 12.031–14.288, P* < *0.001*), consumed alcohol in this month [*OR* = *7.791, 95%CI: 7.077–8.565, P* < *0.001*; Full adjustment: *Odds Ratio (OR)* = *6.010, 95% CI: 5.439–6.641, P* < *0.001*) than those whose parents prohibited it. Low family economic status, not living with the mother, parents' unclear attitude to their children's drinking, and parental drinking were also the risk factors for adolescent drinking.

**Table 4:** Unadjusted and adjusted logistic regression analyses of family environment factors and alcohol drinking.

		**Model 1**						**Model 2**					
		**B**	**Wald**	**P**	**OR**	**95% CI**		**B**	**Wald**	**Sig**.	**Exp (B)**	**95% C.I.for EXP(B)**	
						**Lower**	**Upper**					**Lower**	**Upper**
**Ever consumed alcohol**													
	fas (low = ref.)												
	high	−0.218	17.196	0.000*	0.804	0.725	0.891	−0.314	30.306	0.000*	0.731	0.653	0.817
	middle	−0.237	28.307	0.000*	0.789	0.723	0.861	−0.275	33.946	0.000*	0.759	0.692	0.833
	Cohabitant (Parents both = ref.)												
	Only Mother	−0.040	0.348	0.555	0.961	0.843	1.096	−0.039	0.286	0.593	0.962	0.835	1.108
	Only Father	0.324	9.041	0.003*	1.383	1.119	1.707	0.264	5.180	0.023*	1.303	1.037	1.636
	Other	0.274	27.702	0.000*	1.315	1.188	1.457	0.145	6.513	0.011*	1.157	1.034	1.293
	Parental attitudes favorable toward drinking (Forbid = ref.)												
	Agree	2.713	5202.665	0.000*	15.069	13.998	16.222	2.545	3979.170	0.000*	12.741	11.772	13.789
	Not sure	0.946	345.848	0.000*	2.576	2.331	2.846	0.856	244.331	0.000*	2.353	2.113	2.619
	Father's education level (graduate school = ref.)												
	Secondary and lower	0.311	5.881	0.015*	1.365	1.061	1.755	0.168	1.569	0.210	1.182	0.910	1.537
	University degree	0.073	0.357	0.550	1.076	0.846	1.369	0.039	0.095	0.758	1.040	0.810	1.336
	Mather's education level (graduate school = ref.)												
	Secondary and lower	0.374	6.606	0.000*	1.454	1.093	1.933	0.291	3.598	0.058	1.338	0.990	1.807
	University degree	0.049	0.121	0.728	1.050	0.798	1.381	0.140	0.902	0.342	1.150	0.862	1.535
	Father's drinking frequency (0 = ref.)												
	1~4 times/month	0.128	9.155	0.002*	1.137	1.046	1.235	0.175	14.950	0.000*	1.191	1.090	1.302
	> 4 times/month	0.382	68.015	0.000*	1.465	1.338	1.604	0.394	62.405	0.000*	1.482	1.344	1.634
	Mather's drinking frequency (0 = ref.)												
	1~4 times/month	0.358	70.711	0.000*	1.430	1.316	1.555	0.354	61.226	0.000*	1.424	1.304	1.556
	> 4 times/month	0.575	25.661	0.000*	1.777	1.422	2.219	0.504	17.434	0.000*	1.656	1.307	2.098
**Consumed alcohol within a year**													
	fas (low = ref.)												
	high	−0.302	28.247	0.000*	0.739	0.661	0.826	−0.440	51.613	0.000*	0.644	0.571	0.726
	middle	−0.236	24.637	0.000*	0.790	0.720	0.867	−0.302	36.222	0.000*	0.739	0.670	0.816
	Cohabitant (Parents both = ref.)												
	Only Mother	−0.078	1.130	0.288	0.925	0.801	1.068	−0.075	0.920	0.337	0.927	0.795	1.082
	Only Father	0.360	9.843	0.002*	1.434	1.145	1.796	0.256	4.299	0.038*	1.291	1.014	1.644
	Other	0.236	17.329	0.000*	1.266	1.133	1.415	0.111	3.284	0.070	1.118	0.991	1.260
	Parental attitudes favorable toward drinking (Forbid = ref.)												
	Agree	2.806	4675.566	0.000*	16.544	15.265	17.929	2.573	3442.463	0.000*	13.111	12.031	14.288
	Not sure	1.067	326.743	0.000*	2.905	2.588	3.262	0.959	233.753	0.000*	2.608	2.307	2.949
	Father's education level (graduate school = ref.)						5.819	0.054		
	Secondary and lower	0.447	10.524	0.001*	1.563	1.193	2.047	0.321	5.005	0.025*	1.379	1.041	1.828
	University degree	0.220	2.781	0.095	1.246	0.962	1.613	0.218	2.528	0.112	1.244	0.951	1.627
	Mather's education level (graduate school = ref.)						0.826	0.662		
	Secondary and lower	0.143	0.838	0.360	1.154	0.850	1.566	0.098	0.355	0.551	1.103	0.799	1.522
	University degree	−0.068	0.203	0.652	0.934	0.696	1.255	0.041	0.067	0.795	1.042	0.764	1.420
	Father's drinking frequency (0 = ref.)												
	1~4 times/month	0.177	14.268	0.000*	1.193	1.089	1.308	0.211	18.050	0.000*	1.235	1.121	1.362
	> 4 times/month	0.491	95.036	0.000*	1.634	1.481	1.804	0.498	85.242	0.000*	1.646	1.481	1.829
	Mather's drinking frequency (0 = ref.)												
	1~4 times/month	0.459	106.431	0.000*	1.582	1.450	1.726	0.488	107.659	0.000*	1.629	1.486	1.786
	> 4 times/month	0.586	27.043	0.000*	1.797	1.441	2.242	0.563	22.193	0.000*	1.756	1.389	2.219
**Consumed alcohol within a month**													
	fas (low = ref.)												
	high	−0.368	42.830	0.000*	0.692	0.620	0.773	−0.411	46.725	0.000*	0.663	0.589	0.746
	middle	−0.229	24.882	0.000*	0.796	0.727	0.870	−0.254	27.732	0.000*	0.776	0.706	0.853
	Cohabitant (Parents both = ref.)												
	Only Mother	−0.020	0.072	0.788	0.980	0.847	1.135	−0.060	0.566	0.452	0.942	0.806	1.101
	Only Father	0.435	17.705	0.000*	1.545	1.262	1.892	0.305	7.608	0.006*	1.356	1.092	1.684
	Other	0.241	19.410	0.000*	1.272	1.143	1.416	0.148	6.380	0.012*	1.160	1.034	1.301
	Parental attitudes favorable toward drinking (Forbid = ref.)												
	Agree	2.053	1805.895	0.000*	7.791	7.087	8.565	1.793	1239.180	0.000*	6.010	5.439	6.641
	Not sure	0.927	153.669	0.000*	2.528	2.183	2.927	0.832	115.307	0.000*	2.298	1.974	2.675
	Father's education level (graduate school = ref.)												
	Secondary and lower	0.409	8.338	0.004*	1.505	1.140	1.986	0.375	6.373	0.012*	1.455	1.088	1.947
	University degree	0.202	2.214	0.137	1.224	0.938	1.599	0.250	3.072	0.080	1.284	0.971	1.699
	Mather's education level (graduate school = ref.)												
	Secondary and lower	−0.032	0.042	0.838	0.968	0.712	1.317	0.039	0.056	0.813	1.040	0.753	1.436
	University degree	−0.081	0.285	0.594	0.922	0.685	1.241	0.054	0.115	0.734	1.055	0.773	1.441
	Father's drinking frequency (0 = ref.)												
	1~4 times/month	0.412	66.301	0.000*	1.509	1.367	1.666	0.458	73.895	0.000*	1.580	1.424	1.754
	> 4 times/month	0.614	135.938	0.000*	1.847	1.666	2.048	0.608	118.636	0.000*	1.838	1.647	2.050
	Mather's drinking frequency (0 = ref.)												
	1~4 times/month	0.567	185.406	0.000*	1.763	1.625	1.913	0.640	209.845	0.000*	1.896	1.739	2.067
	> 4 times/month	0.749	63.953	0.000*	2.115	1.760	2.541	0.711	51.904	0.000*	2.036	1.678	2.471

* *P < 0.05. OR, odds ratio; CI, confidence interval; Model 1, Non–adjusted model; Model 2, Adjusted for age, gender, city, location and smoking*.

The relationship between different dimensions of family environment and getting drunk is shown in Table 5, low educational level, and parental alcohol consumption are the risk factors for adolescent drunkenness. Parents' positive attitudes toward children's drinking were also associated with higher risk of getting drunk (*OR* = *5.201, 95% CI:4.734–5.714, P* < *0.001*; Full adjustment, *OR* =*3.433, 95% CI: 3.103–3.797, P* < *0.001)*. Low family economic status, not living with the mother, unclear parents' attitude to their children's alcohol consumption were also had association with drunk behavior significantly.

**Table 5:** Unadjusted and adjusted logistic regression analyses of family environment factors and getting drunk.

	**Model 1**							**Model 2**					
	**B**	**Wald**	***P***	**OR**	**95% C.I**.		**B**	**Wald**	***P***	**Exp(B)**	**95% C.I**.	
					**Lower**	**Upper**					**Lower**	**Upper**
**fas (low = ref.)**													
high	−0.07	1.463	0.226	0.932	0.833	1.044	−0.251	15.653	0.000*	0.778	0.687	0.881
middle	−0.145	8.704	0.003*	0.865	0.785	0.952	−0.235	19.684	0.000*	0.791	0.713	0.877
**Cohabitant (Parents both = ref.)**													
Only Mother	0.128	2.927	0.087	1.137	0.982	1.316	0.091	1.265	0.261	1.095	0.935	1.284
Only Father	0.596	33.911	0.000*	1.815	1.485	2.218	0.496	19.64	0.000*	1.643	1.319	2.046
Other	0.379	47.854	0.000*	1.461	1.312	1.626	0.217	12.954	0.000*	1.242	1.104	1.397
**Parental attitudes favorable toward drinking (Forbid = ref.)**													
Agree	1.649	1180.396	0.000*	5.201	4.734	5.714	1.233	573.843	0.000*	3.433	3.103	3.797	
Not sure	0.632	64.814	0.000*	1.881	1.613	2.193	0.462	31.415	0.000*	1.588	1.351	1.866	
**Father's education level (graduate school = ref.)**													
Secondary and lower	0.269	2.932	0.087	1.309	0.962	1.782	0.049	0.086	0.770	1.05	0.756	1.459	
University degree	−0.04	0.068	0.794	0.961	0.712	1.297	−0.12	0.546	0.460	0.887	0.644	1.220	
**Mather's education level (graduate school=ref.)**													
Secondary and lower	0.352	3.834	0.050	1.422	1	2.024	0.502	6.727	0.009*	1.652	1.13	2.414	
University degree	0.02	0.013	0.909	1.02	0.724	1.439	0.285	2.285	0.131	1.330	0.919	1.924	
**Father's drinking frequency (0 = ref.)**													
1~4 times/month	0.081	2.484	0.115	1.084	0.981	1.198	0.104	3.603	0.058	1.110	0.997	1.235	
> 4 times/month	0.341	41.506	0.000*	1.407	1.268	1.56	0.299	27.215	0.000*	1.349	1.205	1.509	
**Mather's drinking frequency(0 = ref.)**													
1~4 times/month	0.104	5.279	0.022*	1.109	1.015	1.212	0.105	4.616	0.032	1.110	1.009	1.222	
> 4 times/month	0.105	1.029	0.310	1.11	0.907	1.359	−0.053	0.230	0.631	0.948	0.762	1.179	

* *P < 0.05. OR, odds ratio; CI, confidence interval; Model 1, Non–adjusted model; Model 2, Adjusted for age, gender, city, location and smoking*.

## Discussion

This study shows that family environment factors, including parents' attitudes, behavior, and companionship are related to children's drinking behavior. The study supports that family supervision is negatively correlated with adolescent drinking, which is consistent with the results of other foreign studies (19, 28). And this study proved evidence to support this conclusion in the China. Consistent with some research, not living with their mother is a risk factor for adolescent alcohol use, (17, 18). Just as the results of other countries and Taiwan (Province of the People's Republic of China), parental drinking is a predictor of adolescent drinking in this study (21–24). In addition to parents' attitudes, behavior, and companionship, other factors in the family environment also influenced teenagers' drinking behavior in this study. Differ from the research results in some western countries, teenagers from families with low economic status are more likely to drink than those with high economic status (19). The difference may be related to the consumption tendency and culture between different countries.

This study supports the conclusion that family environmental factors are associated with adolescent alcohol consumption. One possible reason is that adolescents connect closely to their parents and internalize their parents' values and norms (29). According to social learning theory, teenagers spend much time with their families, which means teenagers are willing to learn the behavior from the people around them. Social control theory points that parental control can protect children (30, 31).

Previous studies have looked more closely at environmental factors associated with fruit and vegetable intake, calorie intake, fat intake, and water drinking behavior (10). There are few studies on the influence of school/family environment on drinking behavior. Exploring the relationship between family environment and drinking behavior is helpful in finding out which family environment factors are closely related to adolescent drinking behavior. As with other research into the food environment, this is critical for the successfully designing, implementing, and adopting appropriate interventions and policies (32). Teenagers are susceptible to social influences. They are less influenced by their families and become more independent decision-makers. However, at the same time, they tend to have lower levels of self-control, higher levels of impulsivity (33), and higher rates of alcohol use and related harmful behaviors at this stage of life (1, 2, 7–9). Moreover, health behaviors during adolescence impact on lifelong physical and mental health (3, 4). In conclusion, it is vital to carry out this study to explore the relationship between family environmental factors and adolescent drinking behavior.

Although some past studies have explored the relationship between family environment and adolescent alcohol consumption, only a few factors have been identified. Various family environmental factors are considered in this study at the same time. Family structures, economic income levels, and parents' attitudes and behaviors are included in the model simultaneously, which is more detailed than other studies. Moreover, the adjusted model has fully considered the influence of different cities, age, gender, urban area, and other factors. With a relatively large sample size, the results of this study represent the actual situation of a considerable number of Chinese teenagers, which is highly convincing.

There are some limitations to this study. Firstly, due to the limitation of data collection, some spatial environmental factors were not included in this study (34, 35). Moreover, the survey was conducted through the self-report questionnaire, which was not accurate in evaluating variables (36, 37). In addition, our research was conducted in six Chinese cities and is not fully representative of the situation in China. Although this study confirmed the correlation between family environmental factors and adolescents' drinking behavior in six Chinese cities, it could not estabilish the causal relationship as a cross-sectional survey. Further studies are needed to verify the results of this study and explore the causal relationship. If causality is established, more targeted interventions can be implemented. Besides, future research can explore suitable food environment assessment tools for drinking in China. Then appropriate interventions and policies can be designed and implemented according to health-related environmental indicators to impact adolescent drinking behavior positively.

Fairness and ethical principles should be considered in intervene programme and policy making. In particular, authorities should pay more attention to low-income groups and left-behind children. In education and health intervention, health educators should pay attention to adolescents' mental health, emphasizing encouragement rather than punishment.

## Data Availability Statement

The raw data supporting the conclusions of this article will be made available by the authors, without undue reservation.

## Ethics Statement

The studies involving human participants were reviewed and approved by the Ethics Committee for Research in Human Subjects of the National Institute for Nutrition and Food Safety, Chinese Center for Disease Control and Prevention. Written informed consent to participate in this study was provided by the participants' legal guardian/next of kin.

## Author Contributions

RC conducted statistical analysis and completed the first draft of the paper, while SL was responsible for the implementation of the project, data cleaning and participated in the writing of the paper. GM was involved in the survey and gave guidance on writing and revising the article. NZ, MZ, and KG participated in the writing, discussion and revision of the article. SD was responsible for the on-site quality control of the project and participated in the research design. JG participated in the implementation of the project and the revision of the article. XH directed the on-site work of the project and participated in the research design. All authors contributed to the article and approved the submitted version.

## Funding

This study was received by Pernod Ricard (China) Trading Co. Ltd. Financial support, Pernod Ricard (China) Trading Co. Ltd. The company did not intervene in implementing the project, data analysis, and results reporting.

## Conflict of Interest

The authors declare that the research was conducted in the absence of any commercial or financial relationships that could be construed as a potential conflict of interest.

## Publisher's Note

All claims expressed in this article are solely those of the authors and do not necessarily represent those of their affiliated organizations, or those of the publisher, the editors and the reviewers. Any product that may be evaluated in this article, or claim that may be made by its manufacturer, is not guaranteed or endorsed by the publisher.
